# Absence of heartbeat in the *Xenopus tropicalis* mutation *muzak* is caused by a nonsense mutation in cardiac myosin *myh6*

**DOI:** 10.1016/j.ydbio.2009.09.019

**Published:** 2009-09-19

**Authors:** Anita Abu-Daya, Amy K. Sater, Dan E. Wells, Timothy J. Mohun, Lyle B. Zimmerman

**Affiliations:** 1 National Institute for Medical Research, The Ridgeway, Mill Hill, London, NW7 1AA United Kingdom; 2 Dept. of Biology and Biochemistry, University of Houston, Houston TX, USA

**Keywords:** Xenopus tropicalis, mutation, heart, cardiac, myh6, valve, trabeculation, sarcomere, myosin, genetic mapping

## Abstract

Mechanisms coupling heart function and cardiac morphogenesis can be accessed in lower vertebrate embryos that can survive to swimming tadpole stages on diffused oxygen. Forward genetic screens in *Xenopus tropicalis* have identified more than 80 mutations affecting diverse developmental processes, including cardiac morphogenesis and function. In the first positional cloning of a mutation in *X. tropicalis*, we show that non-contractile hearts in *muzak (muz)* embryos are caused by a premature stop codon in the cardiac myosin heavy chain gene *myh6*. The mutation deletes the coiled-coil domain responsible for polymerization into thick filaments, severely disrupting the cardiomyocyte cytoskeleton. Despite the lack of contractile activity and absence of a major structural protein, early stages of cardiac morphogenesis including looping and chamber formation are grossly normal. *Muz* hearts subsequently develop dilated chambers with compressed endocardium and fail to form identifiable cardiac valves and trabeculae.

## Introduction

Formation of the heart is highly conserved in vertebrate species. Genes relevant to human cardiac development and disease can be studied in lower vertebrate models whose externally-developing embryos are easily accessible during heart forming stages and survive for several days on passively-diffused oxygen if cardiac function is compromised experimentally. *Xenopus* researchers have combined classical embryological explant and transplant approaches with over- and mis-expression of gene products (Warkman and Krieg, 2007) to examine early steps in heart formation, including specification of the heart field (Sater and Jacobson, 1989), transcriptional regulation of cardiac identity (Evans et al., 1995; Fu et al., 1998; Grow and Krieg, 1998), and signaling pathways underlying cardiac asymmetry (Branford et al., 2000; Hyatt and Yost, 1998; Ramsdell and Yost, 1999). In zebrafish, heart development studies have built on loss-of-function genetic tools, as well as the optical properties of the embryos for microscopy, to analyze cardiac morphogenesis and valve formation (Beis et al., 2005; Sehnert and Stainier, 2002; Stainier, 2001). As teleost fish are the most diverse vertebrates, due in part to the ancestral genome duplication and subsequent shuffling of gene functions (Force et al., 1999; Postlethwait et al., 2000), comparative studies in other models will help identify developmental mechanisms shared broadly among tetrapods. Loss-of-function studies in *X. laevis* have previously been limited to injection of dominant negative constructs (Grow and Krieg, 1998; Shi et al., 2000) and, more recently, antisense morpholino oligonucleotides (Peterkin et al., 2007; Small et al., 2005). Large-scale genetic approaches are impractical in *X. laevis* due to its pseudotetraploid genome and long generation time, but are well-suited to its diploid relative *Xenopus tropicalis*. *X. tropicalis* reaches maturity in a relatively short 4-6 months, and its small, canonically-organized tetrapod genome (1.5×10^9^ bp in 10 chromosomes) is supported by extensive sequence resources including a high-quality draft genome assembly (http://genome.jgi-psf.org/Xentr4/Xentr4.home.html), over one million ESTs, and a meiotic linkage map of Simple Sequence Length Polymorphisms (SSLPs) (http://tropmap.biology.uh.edu/index.html) (Carruthers and Stemple, 2006; Klein et al., 2006; Klein et al., 2002).

In a pilot screen for chemically-induced mutations in *X. tropicalis*, we recovered several phenotypes with decreased cardiac function (Goda et al., 2006). Here we show that the lack of cardiac contractility in the *muzak* mutant is caused by a nonsense mutation truncating the cardiac myosin heavy chain gene *myh6*. Despite this defect in a major structural component of sarcomeres resulting in absence of myofibrils and contractility, looping and chamber formation appear surprisingly normal. *Muz* hearts subsequently display dilated ventricles and atria and malformed endocardium, segments of which appear collapsed with little or no lumen. Later steps in cardiac development, such as valve formation and trabeculation, are not detected, but it is beyond the scope of this study to determine whether these are direct or indirect effects of the mutation. This report describes the first positional cloning of a mutation in *X. tropicalis*.

## Experimental Procedures

### Frog Strains

The original mutagenesis and fertilization to produce mutant founder F1 animals was performed on the *N* (Nigerian) strain (kind gift of Enrique Amaya, Manchester University, United Kingdom); polymorphic crosses used for mapping were generated using the *IC* (Ivory Coast) strain (kind gift of Robert Grainger, University of Virginia, Charlottesville, USA). Mutant and wt embryos used for mapping and phenotyping were generated from a cross of an F2 *muz/*+ *N/IC* female and an F3 *muz/*+ male produced by crossing an F2 *N/IC* female to an *N/PacBio* (wild-caught animals of unknown origin obtained from Pacific Biological Supply, Inc.) male carrying the mlc2GFP transgene.

### Mapping

Gynogenesis was performed as described previously (Goda et al., 2006). AFLP reactions were performed using the AFLP Analysis System I kit (Invitrogen, 10544-013). PCR products were resolved on 6% denaturing acrylamide gels and visualized by autoradiography. SSLP markers were amplified and resolved as described on the tropmap website (http://tropmap.biology.uh.edu/polyprotocol.html). SSLP markers from the meiotic map 040E09, 018E09, and 026G09 can be found on the tropmap database (http://tropmap.biology.uh.edu/) and have the following sequences:
040E09:
F-AAGTTGCCCTAAAGGTAGGC
R-GATTATTGCTCCGAATGTGG
018E09:
F-CTCAATAATCAGGGCATGTAATC
R-GCAGACATAAGCATTGTACCC
026G09:
F-TGAAGTGAAGCACAGCACAG
R-AGGGACTTTTCCAGATCAAG

Bespoke SSLP markers for scaffold 439 were obtained using Tandem Repeat Finder (http://tandem.bu.edu/trf) and Primer3 (http://primer3.sourceforge.net/). Primers for markers in scaffold 439 were as follows:
439.1:
F-TGCCATTTGTATCCCACCTT
R-CCAGGGATGACTTTGACACA
439.3:
F-TGATCTCAGTGCCAGATGCT
R-TGCTCCAGATAGGTGACGTG
439.10:
F-TTTCTCCTGTGGGCAACTTT
R-GTGCTGGTGGAAGGGAAGTA
SSCP439.1
F-GCGCCCTATAGTGAAATCCA
R-GCACAAAATTGCAGGAGGTT
SSCP439.15
F-CCCTGATCAGTCATGGGTTC
R-GTGACATGACAACGCAAACC

Primers to amplify the *muz myh6* genomic fragment containing stop mutation:
F-CTCGAGCAACAAGTGGATGA
R-GCCCACCATAAAATGACCTG

### Whole Mount In Situ Hybridization

Embryos were staged according to Nieuwkoop and Faber. Fixing and WISH were carried out as described previously (Sive et al., 2000).

WISH probes for *myh6* and *myh6.2* were made by cloning RT-PCR products into the PCRII-TOPO vector using the TOPO TA Cloning Kit (Invitrogen, K4600-40). Probes were prepared by linearizing with XhoI and transcribing with SP6. Primers used were:
*myh6*
F-GCTAGAGAAGATTCGCAAGCAG
R-TCCACAATTGCAGTGTTTTCTT
*myh6.2*
F-TCAGACCTGACAGAGCAACTG
R-TCCCCCTCCATCTTCTTTTT

### RT-PCR

RNA was prepared using Trizol (Invitrogen). cDNA was prepared and amplified with the Enhanced Avian HS RT-PCR kit (Sigma HSRT-100) using the following primers:
*myh6*
F-CCAACAAGGGAACTCTGGAA
R-CTGCAGTTTCTCGTTGGTGA
*myh6.2*
F-AACCCTGCTGCTATTCCAGA
R-TCAAGCTTGGCTTTGGATTT
*myh7b*
F-AACTGGACAAGAAGCGGAGA
R-GGTCCATTACCCCTGGAGTT
*myh15*
F-ATTCCTCCTCACGGACCTTT
R-CGCCCACCTAGAGAGAATGA
*myh8*
F-CCGTCTTGATTACGGGAGAA
R-GGGTTTCTTGTTGGTCAGGA
*odc*
F-GCCAGTAAGACGGAAATCCA
R-CCCATGTCAAAGACACATCG

### Immunoblotting

Dissected hearts from st. 40 tadpoles were collected on ice, resuspended in a modified SDS-sample buffer, boiled for 1 minute, resolved by 6% PAGE, transferred to membrane, and immunoblotted as described previously (Ehler et al., 1999)

### Silver Staining

Silver staining of proteins on SDS-PAGE gels was performed according to manufacturer’s instructions using the Silver Stain Plus Kit (Bio-Rad, 161-0449)

### Morpholino injections

Morpholinos were purchased from GeneTools LLC. A total of 12ng of each morpholino was injected into both cells of a two-cell embryo. Morpholino sequences were as follows:
*myh6* translation-blocking morpholino:
TCTGCCATCAGGGCATCACCCATTG
*myh6* morpholino blocking 1^st^ coding exon splice donor:
CTTATAAATGTAATACCTTGCCATC
Control morpholino:
CCTCTTACCTCAGTTACAATTTATA

### Immunohistochemistry

Stage 42 tadpoles were fixed in 1% paraformaldehyde for 1 hour, washed in PBS, blocked in PBS+10% sheep serum, 2mg/ml BSA and 0.2% saponin for 1 hour at room temperature (RT), then incubated with primary antibody in block solution at 4°C overnight, washed in PBS containing 0.2% saponin and incubated in block solution containing Alexa Fluor 488-conjugated anti-mouse IgG secondary antibody (Invitrogen, A21202) for 2 hours at RT. After washing in PBS with 0.2% saponin, the tadpoles were incubated with 1:20 dilution of Alexa Fluor 568 phalloidin (Invitrogen, A12380) in block solution, washed again, then hearts were dissected and visualized with a Zeiss LSM5 Pascal confocal microscope.

### Plastic Sections and 3-D modeling

Embryos were fixed o/n in Bouin’s fixative (BDH Laboratory Supplies,28087 4V), dehydrated in ethanol, embedded in JB-4 resin (Polysciences Inc.), 3μm sections cut with a Leica RM 2165 microtome, and stained with Hematoxylin and Eosin (both Sigma). Sections were visualized on a Zeiss axiocam microscope, serial images were converted into 8bit greyscale stacks and loaded in Amira 3D Visualisation software Mercury Computer Systems, Germany) and heart structures were manually outlined and annotated. 3D models were generated using the surface rendition tool in Amira.

## Results

### The *muzak* mutation affects heart function

Homozygous *muz* embryos were identified by complete lack of cardiac contractility at heart looping stages (Movie S1). Embryonic blood fails to circulate in *muz* tadpoles, and erythrocytes pool in the ventral blood islands where they form. The tadpoles swim normally, indicating that the mutation does not affect skeletal muscle, and other tissues are not visibly affected. By stage 43 (3 days post fertilization), *muz* embryos develop cardiac edema, and absence of heart function persists until at least feeding tadpole stage (5 days post fertilization). No phenotype was observed in heterozygotes, suggesting that the *muz* allele behaves in a simple recessive fashion.

### *Muz* maps to an interval containing cardiac myosin heavy chain gene

When we began linkage studies to identify the gene underlying the *muz* phenotype, no meiotic map was available. In a map-independent initial strategy, bulk segregant pools of DNA from gynogenetic *muz* and wild type siblings were used to obtain a set of Amplified Fragment Length Polymorphism (AFLP (Vos et al., 1995) markers linked to the mutant locus. 5 bands which amplified from wild type but not *muz* DNA (Figure S1A) were extracted, reamplified, sequenced, and placed on the *X. tropicalis* genome assembly in Version 4 scaffolds 554, 91, 567, 289, 158 (http://genome.jgi-psf.org/Xentr4/Xentr4.home.html). The subsequent release of an *X. tropicalis* meiotic map of SSLP markers (http://tropmap.biology.uh.edu) located these scaffolds in a ~12 cM interval on Linkage Group 1 (LG1). Linkage of the mutation to SSLP markers in these scaffolds was confirmed by bulk segregant analysis of pools of mutant and wild type embryos from a conventional cross of heterozygous carrier siblings (see Figure S1B for an example).

**Figure 1:**
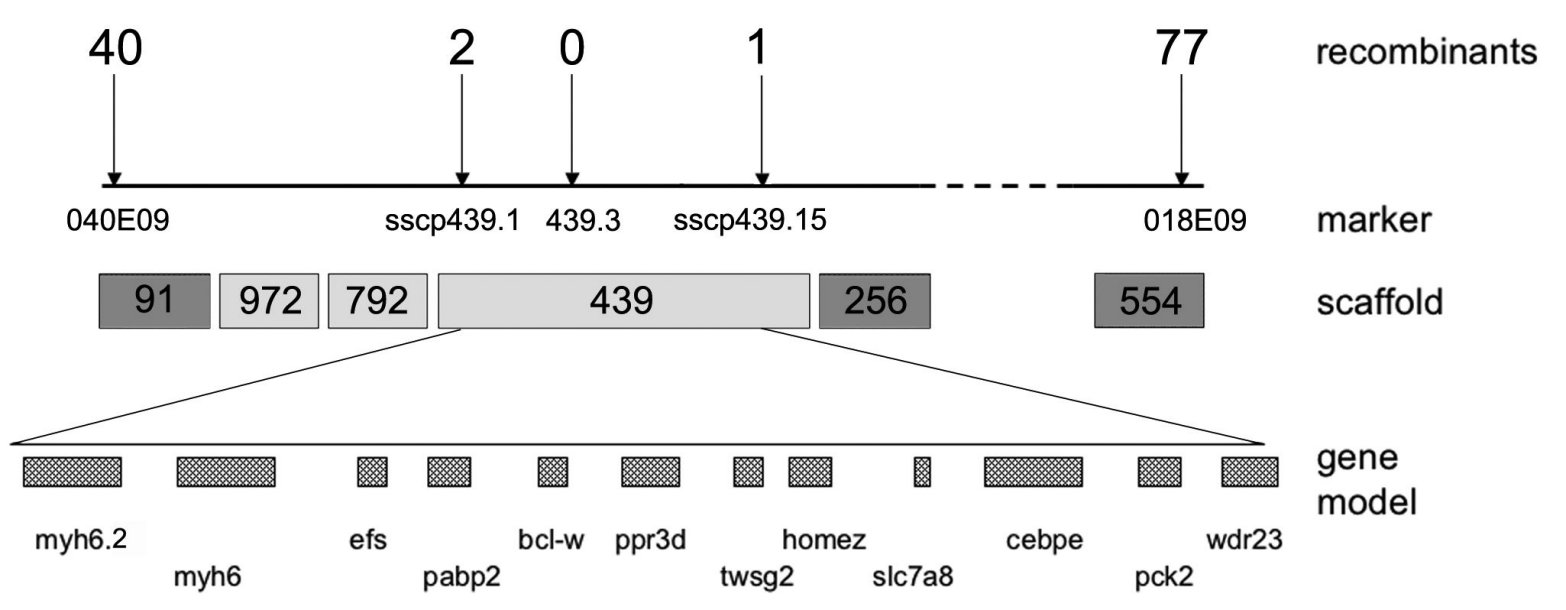
*Muz* maps to an interval containing *cardiac myosin heavy chain* gene A. Individual *muz* embryos were genotyped with SSLP markers from scaffold 91 and scaffold 554. Mapping was refined with SSCP markers (sscp439.1 and sscp439.15) and an SSLP marker (1.439.3) from scaffold 439; number of recombination events detected in 3200 meioses shown above each marker. Dark grey scaffolds are present on the *tropicalis* meiotic map; intervening light grey scaffolds were obtained by analysis of synteny to reference genomes and confirmed by linkage. *Muz* maps to a 370 kb genomic interval between sscp439.1 and sscp439.15 containing 12 gene models in the JGI assembly, including *myh6* and *myh6.2*.

To define the genetic interval containing the *muz* locus, individual *muz* embryos from a conventional sibling cross were genotyped with SSLP markers from LG1 of the meiotic map. Analysis of 3200 meioses placed *muz* between two flanking markers, 040E09 in scaffold 91 (40 recombination events, Figure 1A) and 018E09 in scaffold 554 (77 recombination events). We tested the set of recombinant embryo DNAs further with a marker between the flanking markers, 026G09 (scaffold 256), and found a subset of the recombinants with 018E09 were still recombinant with this polymorphism, whereas all the recombinants with 040E09 were homozygous for the wild type 026G09 allele, suggesting that *muz* was located between the latter two markers. As the X. *tropicalis* genomic sequence assembly was fragmented in this region, and many scaffolds are not represented on the meiotic linkage map, we compared syntenic regions in well-characterized mammalian genomes to generate an *in silico* hypothetical local scaffold assembly. By examining syntenic human and mouse genomic regions that overlapped the termini of scaffolds 256 and 91, we identified candidate intervening scaffolds 439, 792 and 972 in the *muz* interval. Analysis of SSLP markers 1.439.1 (two recombination events), 1.439.3 (no recombination events) and 1.439.10 (1 recombination event) confirmed this local assembly and placed the mutation in scaffold 439. Further analysis refined the *muz* interval to a 370kb region between Single Strand Conformation Polymorphism (SSCP) markers SSCP439.1 (two recombination events) and SSCP439.15 (one recombination event) on scaffold 439 containing 12 gene models on the JGI assembly (Figure 1A and Table S1). The sequence interval containing *muz* was then inspected for candidate genes.

**Figure 2:**
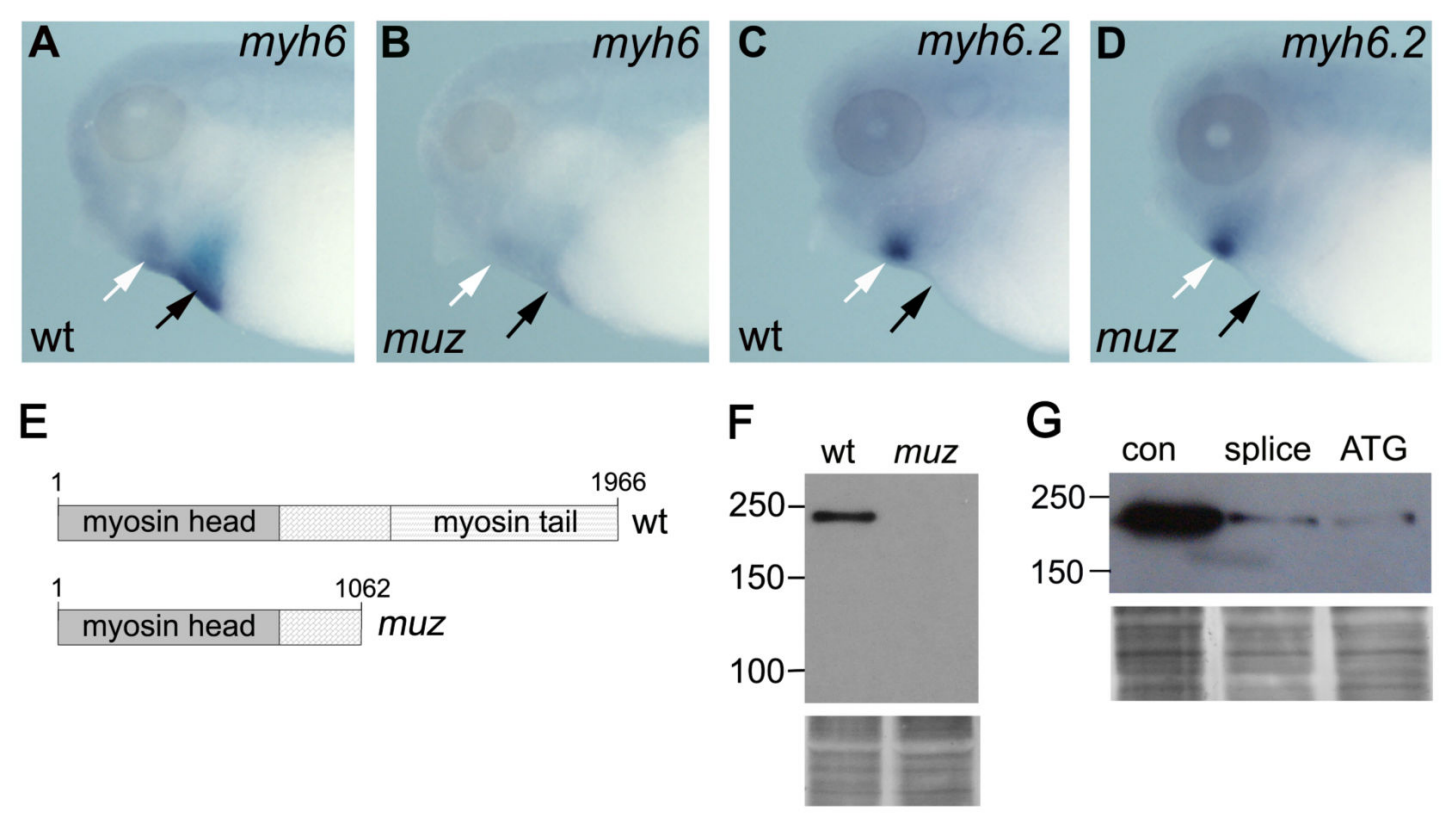
*muzak* is encoded by *myh6* WISH shows *myh6* expression in wild type heart (A, black arrow) and jaw muscle (white arrow) is diminished in *muz* (B). (C, D) *myh6.2* is expressed in jaw muscle (white arrow) but not heart (black arrow), and is unaffected by the mutation. (E) Schematic showing domain structure of wild type *X. tropicalis* myh6 and the truncated protein lacking the myosin coiled-coil tail encoded by the *muz* allele. (F) Western blot analysis does not detect sarcomeric MHC protein in extracts of *muz* heart; silver stained loading control below. (Movie S2 and G) *myh6* morphant hearts do not beat and show strong depletion of sarcomeric MHC protein relative to control morpholino-injected tadpoles; silver stained loading control below.

Compellingly, two gene models in this interval, *myh6* and *myh6.2*, were annotated as myosin heavy chain (MHC), with >88% identity to the human cardiac MYH6 and MYH7 proteins, the major MHC genes expressed in mammalian hearts. These genes are known to be required for normal heart function in humans, with mutations in *MYH6* and *MYH7* implicated in atrial-septal defects and familial hypertrophic cardiomyopathies respectively (Ching et al., 2005; Geisterfer-Lowrance et al., 1990). In human, mouse, and rat these gene pairs are chromosomally adjacent, and are thought to have arisen by tandem duplication before these species diverged, some 70 million years ago (Mahdavi et al., 1984; Mahdavi et al., 1982). Of the two *X. tropicalis* MHC genes on scaffold 439, the centromere-proximal is orthologous to *MYH6* based on mutual best BLAST as well as its strong expression in wild type hearts (Figure 2A, black arrow); weaker expression is also seen in jaw muscles (Figure 2A, white arrow). The distal gene, annotated *myh6.2*, is expressed in developing jaw muscle but not heart (Figure 2C), and hence is unlikely to be responsible for the *muz* phenotype.

To assess whether a defect in *myh6* might underlie the *muz* phenotype, we sequenced cDNA from mutant and unrelated wild type embryos, and found a C to T transition creating a premature stop codon at position 3187 of the coding sequence. Genomic DNA from adult *muz* carrier animals was also found to be heterozygous for this lesion. The resulting truncated protein (1062 aa vs 1996 aa wild type, Figure 2E) is likely to be nonfunctional as it deletes the coiled-coil tail required for dimerization and aggregation into functional thick filaments.

### *Myh6* expression is strongly reduced in *muzak* hearts

We then evaluated how the mutation affected expression of the two MHC genes in the interval. Whole Mount In Situ Hybridization (WISH) showed a significant decrease in *myh6* expression in *muz* embryos compared to wild type (Figure 2A, B), possibly due to nonsense-mediated decay (Peltz et al., 1993; Whitfield et al., 1994). Expression of the neighboring paralog *myh6.2* in jaw muscle was unaffected by the mutation (Figure 2C, D, black arrow).

Levels of cardiac MHC protein were assayed by immunoblotting with the A4.1025 antibody, which recognizes an epitope shared by sarcomeric myosin heavy chain head domains (Dan-Goor et al., 1990) retained in the *muz* allele. A band of ~220kDa is observed in extracts of dissected wild type but not *muz* hearts (Figure 2F). The mutant protein of predicted size ~120kDa is not detected, possibly due to depletion of the mRNA by nonsense-mediated decay, as suggested by WISH. Given the deletion of the tail domain required for thick filament formation and the severe reduction in expression levels, *muz* is likely to be a strong hypomorph or null allele of *myh6*.

### *Myh6* antisense morpholinos phenocopy the *muz* mutation

To confirm that a defect in *myh6* could produce the *muz* cardiac phenotype, we designed morpholino antisense oligonucleotides to deplete the endogenous protein. Both translation-blocking and splice-blocking morpholinos, when injected into both blastomeres of a two-cell embryo, affected cardiac contractility with high penetrance (76/79 and 94/100 injected embryos respectively). In contrast, heart looping and chamber formation were unaffected. Approximately 50% of myh6-depleted embryos had no detectable heartbeat, mimicking the *muzak* phenotype, while the remainder exhibited faint twitching insufficient for blood circulation (Movie S2). Injected embryos were otherwise morphologically normal, with tadpole motility unaffected, indicating that the morpholinos did not interfere with off-target skeletal MHCs. Control morpholino injections had no effect on cardiac function (85/85 wild type). Knockdown efficacy was assayed by immunoblotting protein extracts from dissected morphant hearts with the A4.1025 antibody. Both *myh6* morpholinos strongly depleted cardiac MHC compared to control morpholino (Figure 2G). These gene knockdown data confirm a requirement for *myh6* in cardiac function, strongly supporting the conclusion that a defect in this gene underlies the *muz* phenotype.

### Myh6 is the major cardiac sarcomeric MHC at swimming tadpole stages and is necessary for myofibril formation

**Figure 3:**
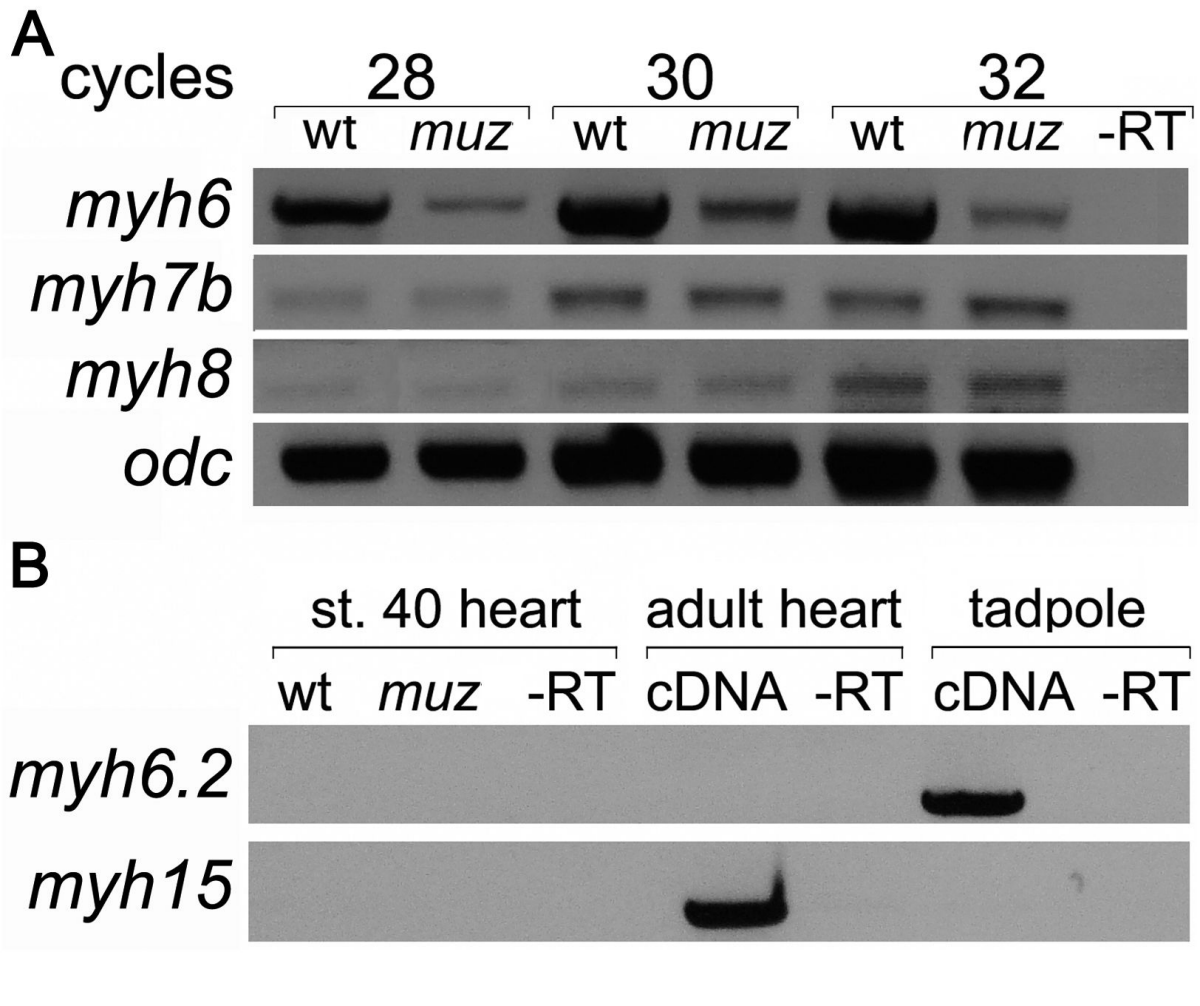
MHC genes expressed in stage 40 wild type and *muz* hearts RT-PCR from isolated stage 40 hearts shows lower levels of *myh6* in *muz*; *myh7B* and *myh8* are unaffected. (A) *myh6.2* mRNA is not detected in wild type or mutant tadpole hearts or wild type adult heart, although it is amplified from whole-embryo mRNA; *myh15* is expressed in adult but not stage 40 tadpole heart(B).

Myh6 is likely to be the principal functional sarcomeric MHC in tadpole hearts, based both on the failure of the A4.1025 antibody to detect any immunoreactive species in *muz* heart extracts and the penetrance of the morphant phenotype. However, since the antibody may not recognize all *Xenopus* MHC proteins, and some morpholino-injected embryos retained faint twitching, we asked whether other sarcomeric MHC mRNAs were expressed in stage 40 hearts or upregulated in *muz*. RT-PCR of dissected stage 40 hearts confirms that *myh6* is expressed strongly in wild type and at much reduced levels in *muz* hearts (Figure 3A). *Myh6.2* was amplified from stage 40 whole embryo mRNA, consistent with its expression in jaw muscle, but not from wild type or *muz* embryonic hearts, nor from adult heart (Figure 3B). The *Xenopus* genome is not thought to contain an ortholog of mammalian *MYH7* (Garriock et al., 2005), and it is likely that *myh6.2* derives from a separate tandem duplication from the one which gave rise to mammalian *MYH6* and *MYH7*. A third cardiac MHC, *myh15/vMHC* (an inactive pseudogene in human), has been found in chicken (Oana et al., 1998), as well as *X. laevis* (Garriock et al., 2005) where it is not expressed until after chamber formation. We found no *myh15/vMHC* expression in hearts of either wild type or *muz* stage 40 embryos by RT-PCR, although it is detected in adult heart (Figure 3B), consistent with previously described onset of expression in *X. laevis* at stage 43. Similarly, no expression in embryonic heart was observed for the skeletal MHCs *myh1,2,3* or *4* (data not shown). However, two MHCs present in mammalian heart EST collections, the slow-tonic *myh7B* and *myh8*, were detected at comparable levels in both wild type and *muz* dissected hearts (Figure 3A). Absence of myh6 protein in *muz* does not appear to induce expression of non-cardiac MHCs or up-regulate *myh7B* and *myh8* mRNAs which, although present in *muz* hearts, are not sufficient to rescue the phenotype.

**Figure 4:**
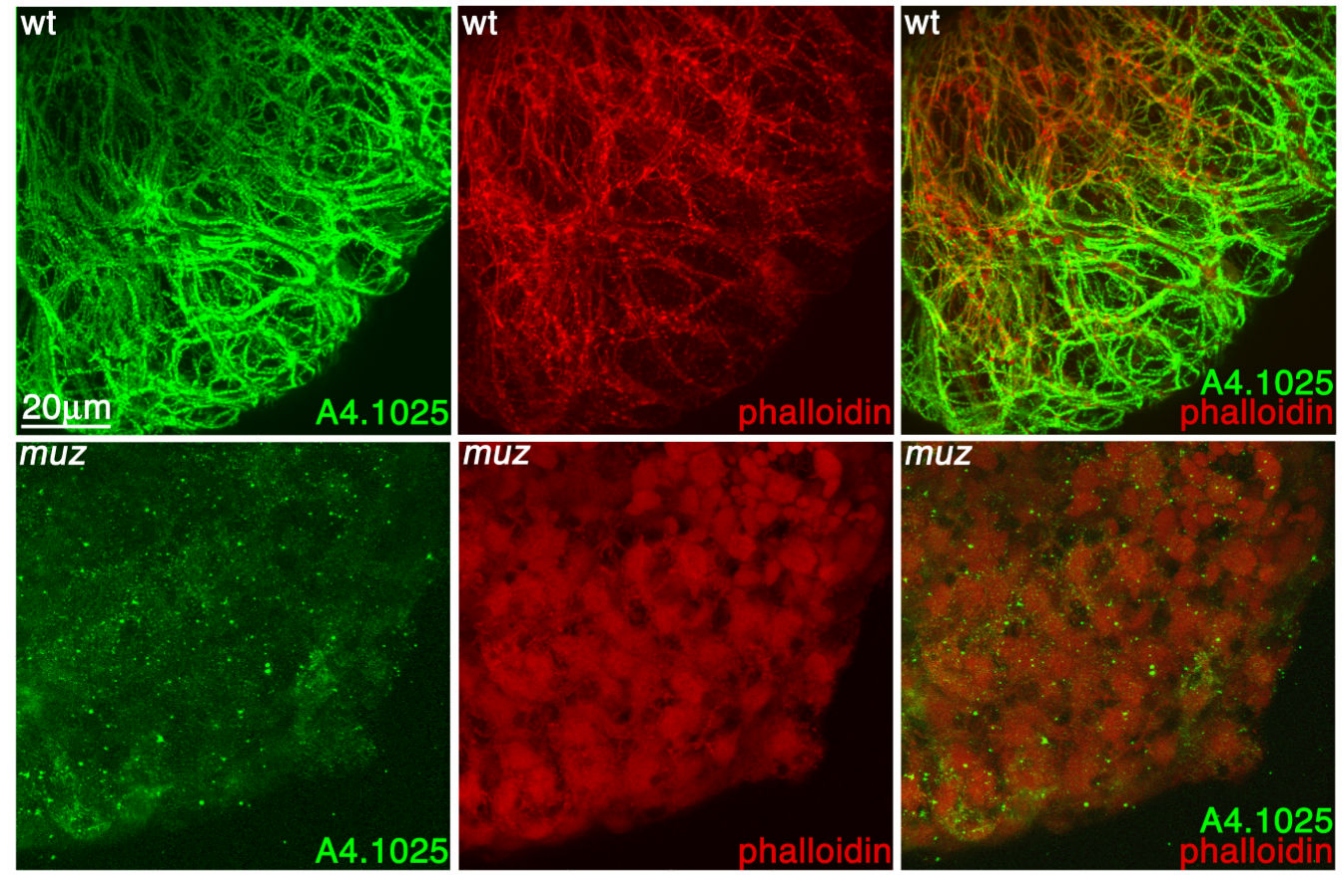
*Muz* hearts lack myofibrils 3D confocal projections of wild type (A) and *muz* (B) hearts immunostained with the pan-sarcomeric MHC A4.1025 antibody (green) and counterstained with phalloidin (red). In wild type hearts, MHC and actin colocalize to myofibrils, while *muz* hearts show very little A4.1025 immunostaining and no fibrillar structures.

We then examined sarcomere formation in *muz* to see whether the remaining myh7B and myh8 could organize myofibrillar structures. Stage 42 wild type and *muz* embryos were stained with the A4.1025 antibody, counterstained with phalloidin, and their hearts dissected and visualized by confocal microscopy. Consistent with the depletion of *myh6* mRNA and protein levels, anti-MHC immunostaining is greatly diminished in *muz* and is not organized in striated myofibrils (Figure 4). Significantly, phalloidin staining shows that actin does not form myofibrils in the mutant heart confirming the lack of any cardiac MHC proteins capable of assembling into sarcomeres in *muz* embryos. Myofibrils were absent at stage 35 (data not shown) when contractions begin as well as stage 40, making it unlikely that *muz* hearts have sarcomeres at any stage of development.

### Cardiac chamber morphology and valve development in *muzak*

In addition to depletion of the myh6 protein, a major structural component of myocardial cells, the *muz* mutation results in abrogation of contractile activity (thought to be required for various steps in cardiac morphogenesis, as well as loss of sarcomeres (known to play signaling as well as mechanical roles in cardiac *function* (Nicol et al., 2000)). We wished to describe how these deficits affect the major morphogenetic steps in heart development.

As in other vertebrates, the *Xenopus* heart initially forms as a linear cardiac tube comprising a muscular myocardial layer surrounding an inner endocardial channel. After undergoing rightward looping, this tube balloons out into chambers separated by cardiac valves. The final stages of heart development in *Xenopus* include trabeculation of the ventricular myocardium and septation of the atrium into two chambers (Kolker et al., 2000; Mohun et al., 2000). To characterize how these processes are affected by absence of myh6 and the resulting lack of sarcomeres and contractility, *muz* hearts were subjected to histological analysis. Plastic sections of the cardiac region of wild type and mutant embryos were obtained at stages relevant to specific tissue formation processes: stage 35 (heart looping), 40 (onset of chamber formation), and 42 (valve formation).

**Figure 5:**
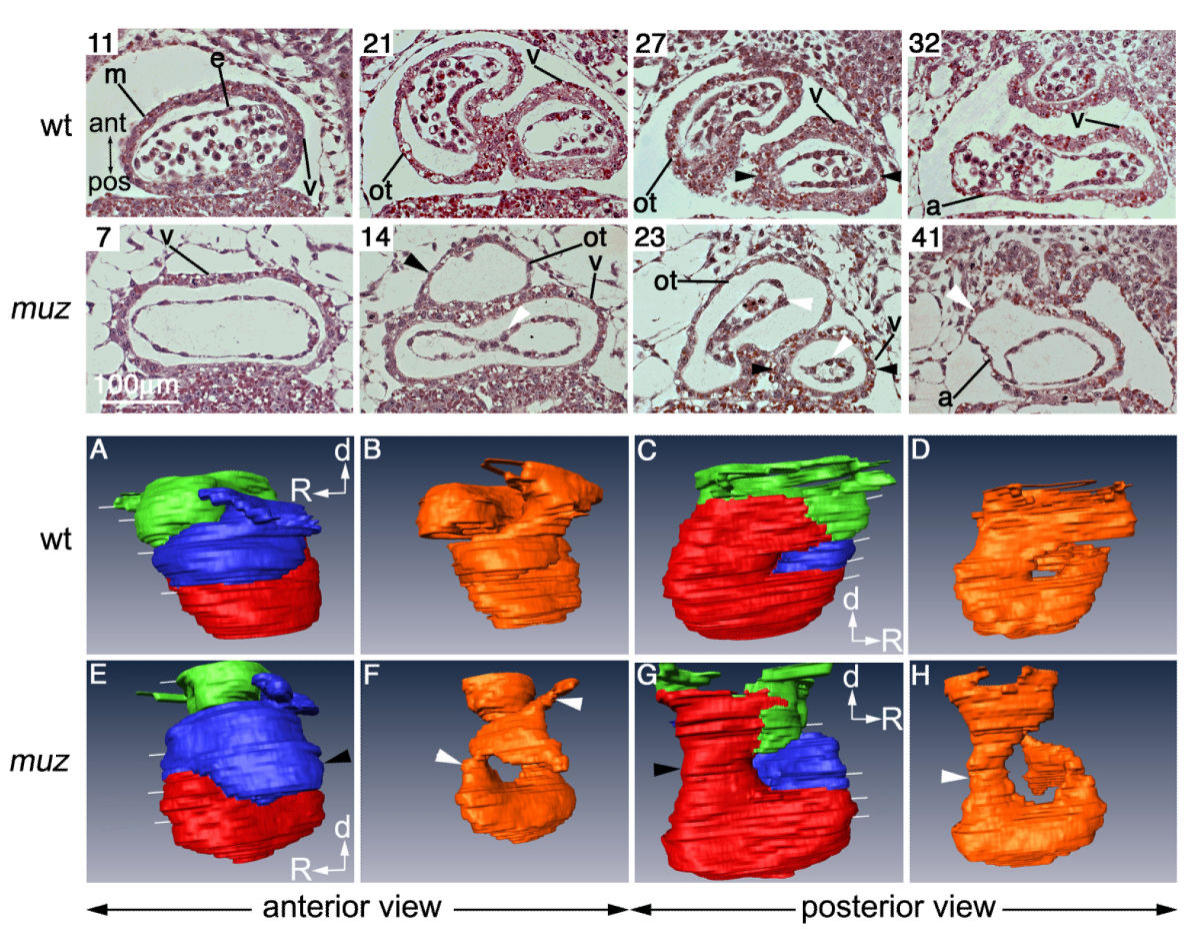
Altered chamber morphology in *muz* hearts Coronal plastic sections of stage 40 wild type and *muz* hearts (top rows) numbered from ventral side of cardiac cavity, and indicated by white lines in 3D models (bottom rows). m= myocardium, e= inner endocardial tube, v= ventricle, ot= outflow tract , a= atrium. No blood cells are seen in the *muz* sections due to lack of circulation, and myocardial layer appears thinner throughout the *muz* heart compared to wild type. The *muz* ventricle is wider than in wild type (sections 7 and 11), while outflow tract and atrium are dilated (sections 14, 23 and 41). Abnormal *muz* chamber morphology is highlighted in 3D projections of outlines of myocardium (A, C, E, G, red=ventricle, blue=outflow tract, green=atrium) and endocardium (B, D, F, H, orange), including elongated ventricle, dilated outflow tract (black arrowhead in E) and narrow cardiac tube at AVC level (black arrow in G). *muz* endocardium is very compressed with drastically reduced lumen (white arrows in 23, F and H)

Figure 5 shows stage 40 wild type and *muz* hearts; sections are numbered to indicate their position in the stack beginning at the ventral side of the cardiac cavity. Outlines of myocardial and endocardial layers in the image stacks were then used to generate 3D projections (Figure 5A-H, see also movies S3, S4 for a rotating view). Regions of the heart are indicated by colour: the myocardium of the outflow tract (blue) was defined by morphological position; thinner myocardium in dorsal sections (green) forms the atrium; thicker myocardium (red) in ventral sections is clearly ventricular (e.g. Figure 5 section 32 ‘v’); however, in malformed mutant hearts, where atrial and ventricular chambers showed little difference in wall thickness, the precise border was assigned arbitrarily.

Cardiac chambers in *muz* are dilated, and at stage 40 the myocardial wall appears thinner than wild type throughout. Segments of the endocardial tube, notably in outflow tract and atrioventricular canal (AVC), appear constricted with little lumen (white arrowheads, section 23 and F, H). The expanded ‘peri-endocardial’ region between the distended myocardium and the constricted endocardium distorts their alignment (white arrowhead, section 14). The cardiac tube at AVC level, spanned by black arrowheads in sections 27 and 23, is narrower in the mutant (black arrowhead in C and G). Dorsally the *muz* atrium is usually distended (white arrowhead, section 41). No blood cells are seen in *muz* hearts at this stage due to lack of circulation. Many of these abnormalities are already present prior to chamber differentiation in earlier looped cardiac tube (stage 35) *muz* tadpoles, including the dilated outflow tract, collapsed endocardial tube, and the narrow cardiac tube at the level of the AVC (Figure S2).

**Figure 6:**
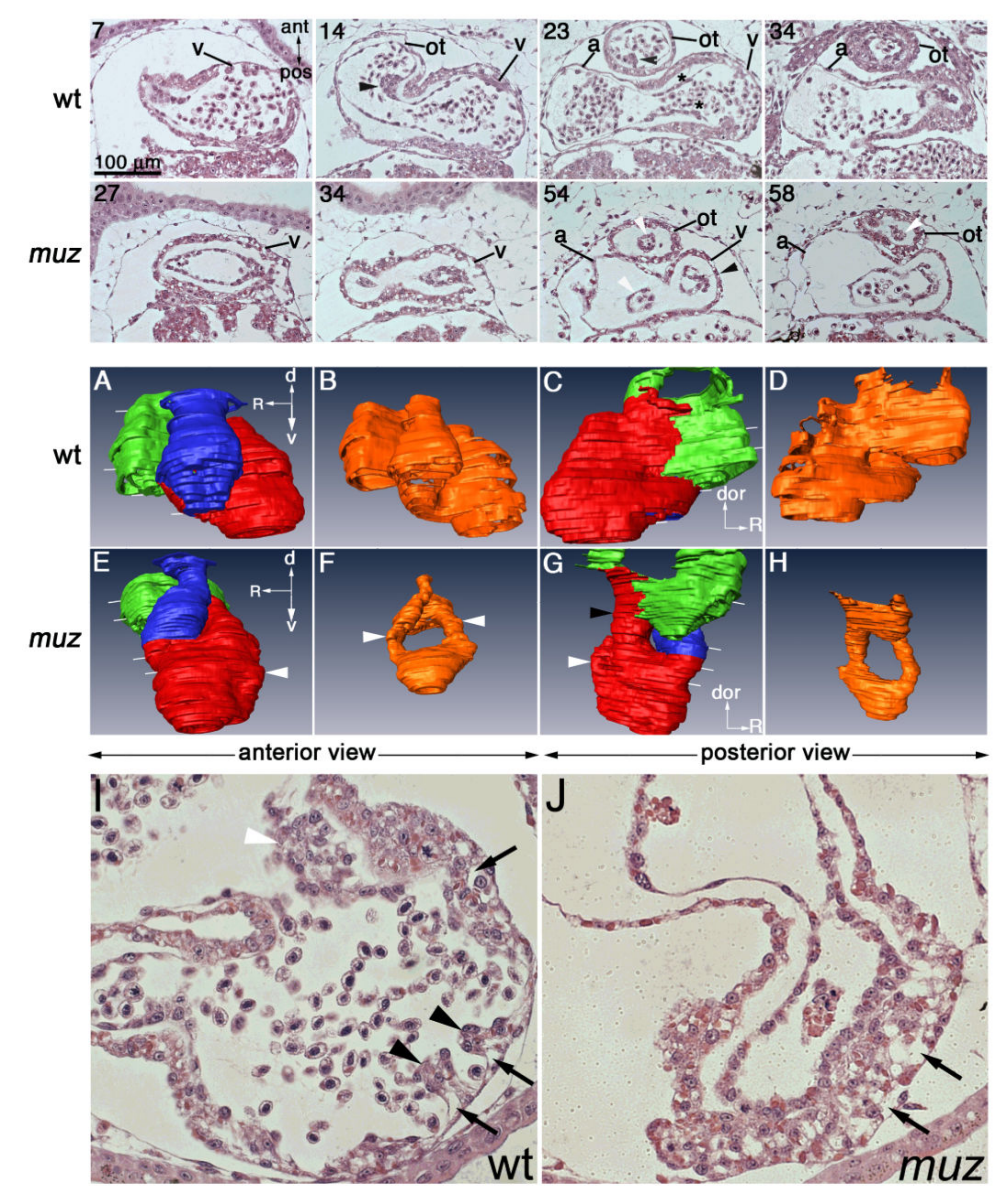
*Muz* hearts become dilated and lack valves and trabeculae Coronal plastic sections of stage 42 wt and *muz* hearts (top rows) numbered from ventral side of cardiac cavity, and indicated by white lines in 3D models (middle rows). v= ventricle, ot= outflow tract , a= atrium. Wild type hearts show a spiral valve in the outflow tract (sections 14, 23, black arrows), and thickening of endocardium preceding atrioventricular valve formation (section 23, black asterisk). Valve formation is not detected in *muz* hearts, and endocardial lumen is drastically reduced in outflow tract and AVC regions (white arrowheads sections 54, 58, also compare models B and F). Endocardial cushion formation in AVC can also be seen in transverse sections of stage 42 wild type (I, white arrowhead) hearts but not in *muz* (J). Trabeculation has initiated in the wild type ventricle (I, black arrowheads) but is absent in *muz* (J). At this stage the ventricular myocardium has a vacuolated appearance in both wt and mutant embryos (I, J black arrows). Middle two rows: 3D projections of outlines of myocardium (A, C, E, G) and endocardium (B, D, F, H) highlight abnormal *muz* chamber morphology; red = ventricle, green = atrium, blue = outflow tract, orange = endocardium. *Muz* ventricles are elongated relative to wild type (E, G white arrows). A narrow tube connects *muz* ventricle and atrium (section 54 and G, black arrowheads; compare to 23, C).

At slightly later stages, valve formation begins in *Xenopus*; this process is not thought to occur in the absence of contraction in zebrafish (Bartman et al., 2004). We therefore examined plastic sections of stage 42 wild type and *muz* tadpoles (Figure 6). A spiral valve can be distinguished in the outflow tract of wild type embryos (black arrowhead, sections 14 and 23), and the ‘endocardial cushion’ valve precursors are forming in the AVC (black asterisks, section 23). In stage 42 *muz* hearts, as at earlier stages, the endocardial tube is often narrower (white arrowheads, sections 54, 58 and F, H) and no valve formation can be discerned. Transverse sections more clearly show endocardial cushions forming in the AVC region of wild type (Figure 6I, white arrowhead) but not *muz* hearts (Figure 6J). Since it is difficult to unambiguously identify valve-forming AVC and outflow tract positions in the morphologically-distorted mutant hearts, we have also examined complete stacks of cardiac-level sections from 10 *muz* embryos without detecting identifiable cushions at any position (data not shown). Endocardial cushions were clearly visible in 10/10 sibling wild type embryos.

Another important process, trabeculation, in which the ventricular myocardium takes on a spongiform appearance, is also occurring at this stage. In wild type hearts, myocardial cells can be seen proliferating and protruding into the lumen (Figure 6I, black arrowheads); interestingly, these cells also take on a vacuolated appearance that may be integral to the mechanism of trabeculation (black arrows). No trabeculae are seen in *muz* ventricular myocardium, which is very thick but retains abundant vacuole-like structures similar to wild type (Figure 6J, black arrows).

3-D modeling at stage 42 reveals that mutant cardiac morphology is becoming progressively more distorted (Figure 6, see also movies S5, S6 for a rotating view). Whereas in wild type the outflow tract rises sharply out of the ventricle towards the dorsal side of the embryo (Figure 6 section 14 and A), in *muz* hearts it gently loops out of the end of the elongated ventricle (Figure 6, 34 and E). The narrow cardiac tube at AVC level seen at stages 35 and 40 becomes more pronounced at stage 42 (Black arrowhead, G). Again, blood cells are absent in the *muz* heart except for a few in the atrium and inflow tract (Figure 6 section 58). The qualitative morphological abnormalities described here are consistently present in *muz* embryos at stages 40-42 (>10 mutant and wild type hearts examined in plastic sections, and 6 mutant and wild type examined by High Resolution Episcopic Microscopy (HREM)(data not shown)).

Analysis of histological sections of *muz* hearts demonstrates that later steps in heart development such as valve formation and trabeculation do not occur in the absence of myh6/contractility and sarcomeres. The morphology of heart chambers is altered; dilated ventricles and atria are observed as early as stage 35, and become progressively more pronounced. The endocardium is likewise severely malformed, with segments of lumen highly constricted. It is beyond the scope of this analysis to conclude that these late effects are direct consequences of the mutation in *myh6*. However, early steps in cardiogenesis, such as looping and chamber formation, are relatively unaffected by absence of contractility and blood flow.

### Discussion

The mapping of *muzak* marks the first identification of a sequence lesion underlying an induced mutation in *X. tropicalis*, an important step in establishing this species as a genetic model organism. The non-contractile heart phenotype is tightly linked to a nonsense mutation in the *myh6* gene deleting the coiled-coil tail domain required for aggregation into functional thick filaments. This nonfunctional peptide, associated with severe reduction of mRNA and absence of detectable MHC protein and myofibrils, suggests that the *muz* allele is a strong hypomorph or null of *myh6*. Loss-of-function studies in *Xenopus* have previously been limited to morpholino knockdown and dominant negative strategies, where it can be difficult to obtain reproducible and complete deletions of specific activities. Precision loss-of-function tools are available in genetic systems such as mice and zebrafish. However, mutational analysis of cardiac development can be challenging in mammals, where heart function is required early in gestation; indeed, the null phenotype of mouse *Myh6* has not been characterized due to early lethality (Jones et al., 1996). Genetic screens in fish have uncovered a large number of cardiac gene functions, but the basic structure of the two-chambered fish heart differs significantly from the four-chambered mammalian heart. The ancestral teleost genome duplication has also led to wholesale reassignment and shuffling of gene functions (Force et al., 1999; Postlethwait et al., 2000), complicating orthology assignment and contributing to the diversity of developmental mechanisms. For example, zebrafish cardiac valves are thought to form by an atypical direct invagination of endocardial epithelia into leaflet structures (Scherz et al., 2008) rather than via a mesenchymal ‘endocardial cushion’ intermediate as has been described in other vertebrates (Armstrong and Bischoff, 2004; Eisenberg and Markwald, 1995) and indeed other fish (Gallego et al., 1997; Icardo et al., 2004). Genetic analysis of *X. tropicalis*, with its more conventionally-organized tetrapod genome and array of functional assays, will help bridge studies of cardiac development from teleost models to amniotes.

In *muzak* embryos, the early processes of heart looping and chamber formation are remarkably successful despite the lack of myh6 protein and consequent absence of myofilaments, sarcomeres, heartbeat and blood flow. We have not ascertained which of these deficits is responsible for the later defects observed in chamber morphology, valve formation, and trabeculation, or whether these are direct or indirect consequences of the mutation. However, it is worth noting that mutant hearts never initiate detectable contraction and beating, and hence develop in the complete absence of blood flow-mediated pressure load and shear stress. The role of mechanical forces in cardiac morphogenesis has been studied extensively, with conflicting results (Taber, 2006). In diverse vertebrates, beating begins substantially prior to requirements for transport of blood-borne oxygen and nutrients, consistent with a role as a physical influence on early steps such as looping and chamber formation (Burggren et al., 2000; Mellish, 1994; Pelster and Burggren, 1996; Territo and Burggren, 1998); indeed, heart looping begins when the first myofibrils appear (Manasek et al., 1978). Mechanical or genetic perturbation of contraction and blood flow have supported a role in these early steps in some cases (Hove et al., 2003; Huang et al., 2003; Nishii et al., 2008), but not in others (Sehnert et al., 2002). Our histological analysis and 3-D modelling of *muz* hearts demonstrates that contractility and blood flow are not required for the key early steps of looping and chamber formation in this tetrapod.

Slightly later in heart development, chamber outgrowth or ‘ballooning’ is thought to be shaped by mechanical forces. Analysis of the chamber-specific MHC mutations *weak atrium* (atrial MHC, *myh6*) and *half hearted* (ventricular MHC, *vmhc*) show that blood flow promotes cardiomyocyte elongation in specific regions of the linear heart tube in the zebrafish embryo, while contractility restricts cell size and elongation (Auman et al., 2007). The *muzak* cardiac tube still undergoes ballooning into ventricle and atrium, suggesting that factors other than fluid shear forces can initiate chamber outgrowth. Another striking feature of *muz* hearts is the constriction of the lumen seen in the atrioventricular canal and outflow tract segments of the endocardial tube. The developing heart has been compared to a specialized blood vessel; arteries are thought to remodel their lumen diameters to maintain shear stress near an optimal set point, decreasing diameter in response to decreased shear (Taber et al., 1995). It is possible that morphogenesis and inflation of these heart regions are particularly shear-dependent.

Another key step in cardiac development, remodeling of the ventricular myocardium to form trabeculae, is critical for increasing the surface area through which the muscle mass of the ventricle can diffuse oxygen prior to the development of coronary circulation (Sedmera, 2005). Trabeculation does not occur in *muz*; instead the non-trabeculating regions of the ventricular myocardial wall become very thick. Wild type myocardium undergoing trabeculation displays a vacuolated appearance that we also observe in *muz*. Failure to form trabeculae could be simply due to lower oxygen requirements of the inactive mutant heart; trabeculation could also depend structurally on sarcomere integrity, or require signals from the overlying endocardium (Gassmann et al., 1995; Grego-Bessa et al., 2007; Meyer and Birchmeier, 1995), some of which regulate myocyte proliferation. Interestingly, the non-trabeculating *muz* myocardial wall appears as thick as its wild type counterpart, suggesting that proliferation may still occur. Although endocardium does not express *myh6*, it is known to alter its gene expression in response to haemodynamic changes (Groenendijk et al., 2005); it remains to be seen whether specific trabeculation signals are affected in the mutant.

As the embryonic heart matures, efficient function depends on the formation of endocardial valves to prevent retrograde blood flow between chambers. Studies in *Danio* suggest that when contraction and/or blood flow is disrupted mechanically (Hove et al., 2003) or genetically (Bartman et al., 2004), valve formation is impaired, but this process is now thought to occur by an atypical mechanism of direct leaflet invagination in zebrafish (Scherz et al., 2008). We have seen no evidence of precursors or differentiated valves in *muz* embryos, consistent with a requirement for blood flow in valve formation mediated by more conventional endocardial cushion intermediates. However, in the absence of cushion-specific markers, which have not been described in *Xenopus*, morphological distortion of the *muz* endocardium makes it difficult for us to conclusively rule out the presence of ectopic cushion precursors.

Several other mutations affecting heart function have been identified in pilot genetic screens in *X. tropicalis* (Goda et al., 2006; Grammer et al., 2005; Noramly et al., 2005), rapid mapping strategies have been established ((Khokha et al., 2009); see also Supplemental Figure S3 for an *X. tropicalis* genetic mapping strategy flowchart), and reverse genetic resources are being developed ((Goda et al., 2006), http://www.sanger.ac.uk/Teams/Team31/xtmr.shtml) from which mutants in known genes can be obtained. Heart development in *X. tropicalis* genetic models can be analyzed with a broad array of molecular, genomic, and embryological tools, including gain-of-function mRNA expression screens (Smith and Harland, 1992) to identify interacting suppressor or enhancer functions and sophisticated explant assays modeling differentiation to diverse tissue fates including beating cardiac muscle (Latinkic et al., 2003). Reinforced by these robust functional assays, genetic approaches in amphibians complement rapidly-advancing genomics technologies for dissecting tetrapod developmental processes. The work presented here demonstrates the feasibility of positionally cloning mutations in *X. tropicalis*, greatly increasing the range of genetic studies.

## Supplementary Material

01 Figure S1. AFLP and SSLP markers define the *muz*-containing interval on LG1. (A) AFLP reactions on bulk segregant wild type and *muz* genomic DNA produced five polymorphic markers linked to the mutation (white boxes). (B, left panel) Linked AFLP markers were placed on genomic sequence scaffolds, from which SSLP markers were tested on bulk segregant (BS) wt and *muz* DNA, confirming linkage to these scaffolds. (B, right panel) Genotyping of individual *muz* embryos with SSLP markers 040E09 and 018E09 defined the *muz*-containing interval. Recombinant embryos are indicated by asterisks.

10 **Movie S6.** 3-D model of a stage 42 *muz* heart rotating about it’s dorsal-ventral axis. Ventral is at bottom. Red=ventricle, blue=outflow tract, green=atrium.

02 **Table S1.** Gene models in the *muz*-containing genetic interval and their official full names as provided by the HUGO gene nomenclature committee.

03 Figure S2. Abnormal morphology of *muz* hearts is evident as early as the looping cardiac tube stage.
Coronal plastic sections of stage 35 wt and *muz* hearts (top rows), numbered from ventral side of cardiac cavity, and indicated by white lines in 3D models (bottom rows). V=ventricle, ot=outflow tract, a=atrium. Bottom two rows: 3D projections of outlines of myocardium (A, C, E, G, red=ventricle, blue=outflow tract, green=atrium) and endocardium (B, D, F, H, orange). Abnormal cardiac morphology is already evident in *muz* hearts at the looped cardiac tube stage. The *muz* ventricle is enlarged (E and G), except at the level of the AVC where a narrow cardiac tube connects the ventricular and atrial chambers (24 and G, black arrowhead). The outflow tract is dilated (25 and E, black arrowhead). The myocardial layer is thinner throughout the mutant heart and the endocardial tubes appear much narrower, with little lumen (24, 39 and F, white arrowheads).

04 Figure S3. Flowchart for Genetic Mapping in *X. tropicalis*
A recessive **mutation** (asterisk) is induced on one strain (for our screens this is an outbred *N (Nigerian)* stock) represented by red chromosomes, top left. Polymorphisms for mapping are introduced by crossing to strain(s) which differ from *N* at many sequence loci (blue chromosomes, top right (for *muz*, depending on availability of appropriate genders for crosses, these included both *IC* and *PacBio*)) to obtain a hybrid **map cross generation**. Meiotic recombination generates crossovers between red and blue strain DNA. In phenotypically mutant embryos, regions close to the homozygous mutant locus are likely to be homozygous ‘red’; with increasing distance, intervening crossovers produce heterozygous red:blue. Rapid assignment of mutations to chromosome/**linkage group** can often be accomplished by analysis of gynogenetic embryos with polymorphic markers from each of the 10 *tropicalis* centromeres (see Khokha et al. 2009); the ratio of mutant to wild type in gynogenetic embryos also provides an estimate of the mutation’s distance from the centromere. Representative chromosomes from two mutant (left) and wild type (right) gynogenetic embryos are shown; linkage is detected to red strain centromere (red circles) of the large chromosome; wild type or unlinked chromosomes show both blue and red centromere alleles. In cases where mutant loci are far from centromeres, mutations can be placed on a linkage group by assaying more distal polymorphisms from the meiotic map (**whole genome marker scanning**), or using the more cumbersome **AFLP** (used for initial steps of *muz* mapping predating the meiotic map (Vos et al., 1995)) to obtain linked sequences in map regions where markers are at low density. For further intermediate- and high-resolution mapping, embryos from natural matings are preferable (right column). To **define the interval** containing the mutation, polymorphisms derived from the meiotic map ~3-10cM apart flanking the locus are identified in mutant embryos: if markers are on opposite sides of the mutation, mutant embryos with crossovers between one marker and the mutant locus will not be recombinant for the marker on the other side and vice versa. High-resolution mapping involves typing a large number (>500) of mutant embryos with the flanking markers to identify the **mutant set containing proximal crossovers**. The small set can then be analyzed with subdividing polymorphisms to refine the interval, ideally placing the mutation on a single sequence scaffold that can be inspected for candidate genes. Candidate genes can be evaluated by expression in affected tissue, sequence lesions in the mutant, and the ability to phenocopy or rescue the mutation.

05 Movie S1. *Muz* embryos have no heartbeat.
Stage 37 *muz* embryo has no cardiac contractility (top), a wt sibling shows a strong regular heartbeat (bottom).

06 Movie S2. Myh6 morpholino phenocopies the *muz* mutation.
An myh6 morpholino injected embryo is morphologically normal but has almost no visible heartbeat, although at high magnification very weak twitching can be seen (bottom). In contrast the control morpholino has no effect on cardiac function (top).

07 **Movie S3.** 3-D model of a stage 40 wild type heart rotating about it’s dorsal-ventral axis. Ventral is at bottom. Red=ventricle, blue=outflow tract, green=atrium.

08 **Movie S4.** 3-D model of a stage 40 *muz* heart rotating about it’s dorsal-ventral axis. Ventral is at bottom. Red=ventricle, blue=outflow tract, green=atrium.

09 **Movie S5.** 3-D model of a stage 42 wild type heart rotating about it’s dorsal-ventral axis. Ventral is at bottom. Red=ventricle, blue=outflow tract, green=atrium.
