# Supplementary figures and images for: Absence of heartbeat in the Xenopus tropicalis mutation muzak is caused by a nonsense mutation in cardiac myosin myh6
Source: Dev Biol. Author manuscript; Available in PMC 2010 Dec 1. (PMC2786259; doi:10.1016/j.ydbio.2009.09.019)

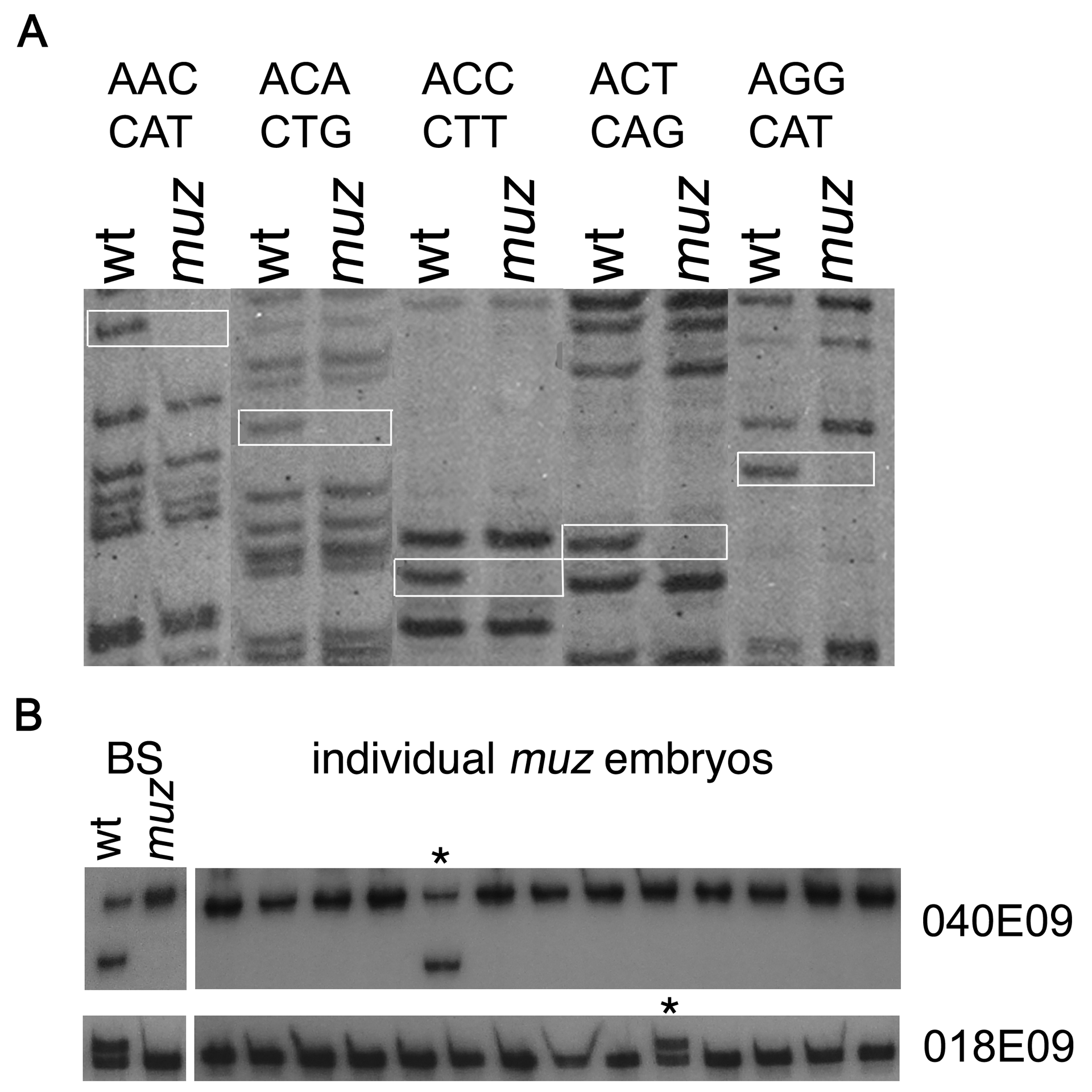

Supplement: 01 — Figure S1. AFLP and SSLP markers define the muz-containing interval on LG1. (A) AFLP reactions on bulk segregant wild type and muz genomic DNA produced five polymorphic markers linked to the mutation (white boxes). (B, left panel) Linked AFLP markers were placed on genomic sequence scaffolds, from which SSLP markers were tested on bulk segregant (BS) wt and muz DNA, confirming linkage to these scaffolds. (B, right panel) Genotyping of individual muz embryos with SSLP markers 040E09 and 018E09 defined the muz-containing interval. Recombinant embryos are indicated by asterisks. [file NIHMS148281-supplement-01.tif]

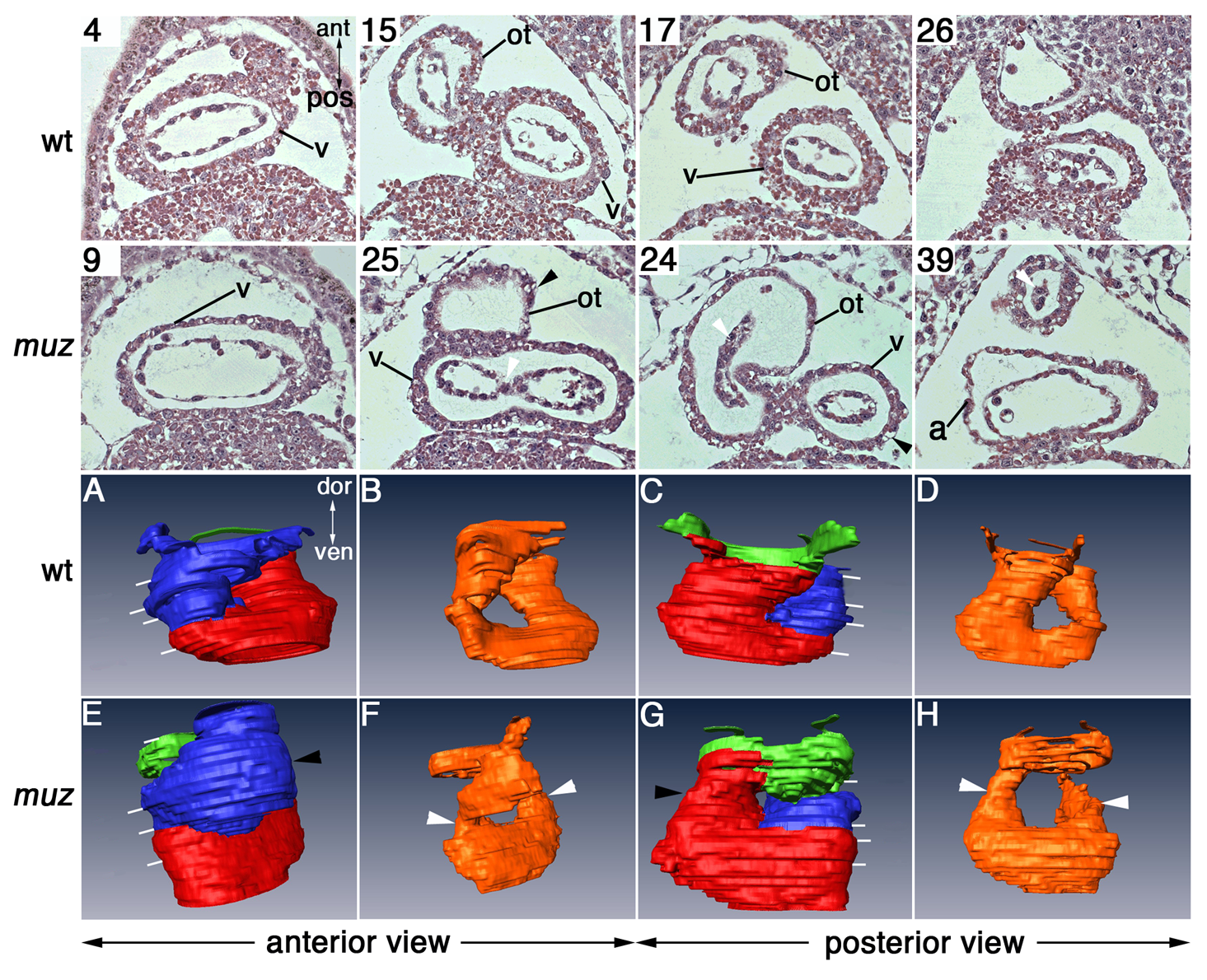

Supplement: 03 — Figure S2. Abnormal morphology of muz hearts is evident as early as the looping cardiac tube stage. Coronal plastic sections of stage 35 wt and muz hearts (top rows), numbered from ventral side of cardiac cavity, and indicated by white lines in 3D models (bottom rows). V=ventricle, ot=outflow tract, a=atrium. Bottom two rows: 3D projections of outlines of myocardium (A, C, E, G, red=ventricle, blue=outflow tract, green=atrium) and endocardium (B, D, F, H, orange). Abnormal cardiac morphology is already evident in muz hearts at the looped cardiac tube stage. The muz ventricle is enlarged (E and G), except at the level of the AVC where a narrow cardiac tube connects the ventricular and atrial chambers (24 and G, black arrowhead). The outflow tract is dilated (25 and E, black arrowhead). The myocardial layer is thinner throughout the mutant heart and the endocardial tubes appear much narrower, with little lumen (24, 39 and F, white arrowheads). [file NIHMS148281-supplement-03.tif]

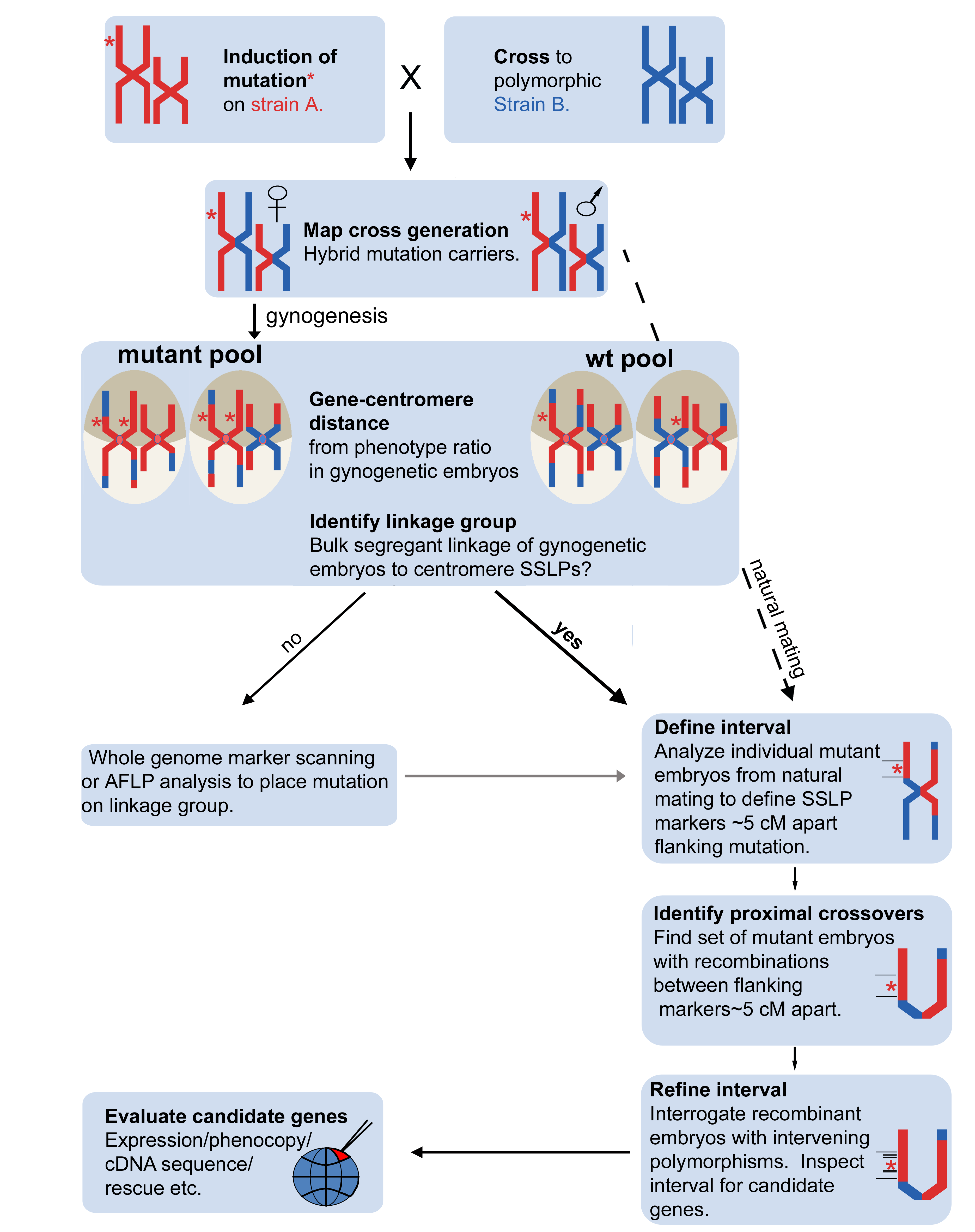

Supplement: 04 — Figure S3. Flowchart for Genetic Mapping in X. tropicalis A recessive mutation (asterisk) is induced on one strain (for our screens this is an outbred N (Nigerian) stock) represented by red chromosomes, top left. Polymorphisms for mapping are introduced by crossing to strain(s) which differ from N at many sequence loci (blue chromosomes, top right (for muz, depending on availability of appropriate genders for crosses, these included both IC and PacBio)) to obtain a hybrid map cross generation. Meiotic recombination generates crossovers between red and blue strain DNA. In phenotypically mutant embryos, regions close to the homozygous mutant locus are likely to be homozygous ‘red’; with increasing distance, intervening crossovers produce heterozygous red:blue. Rapid assignment of mutations to chromosome/linkage group can often be accomplished by analysis of gynogenetic embryos with polymorphic markers from each of the 10 tropicalis centromeres (see Khokha et al. 2009); the ratio of mutant to wild type in gynogenetic embryos also provides an estimate of the mutation’s distance from the centromere. Representative chromosomes from two mutant (left) and wild type (right) gynogenetic embryos are shown; linkage is detected to red strain centromere (red circles) of the large chromosome; wild type or unlinked chromosomes show both blue and red centromere alleles. In cases where mutant loci are far from centromeres, mutations can be placed on a linkage group by assaying more distal polymorphisms from the meiotic map (whole genome marker scanning), or using the more cumbersome AFLP (used for initial steps of muz mapping predating the meiotic map (Vos et al., 1995)) to obtain linked sequences in map regions where markers are at low density. For further intermediate- and high-resolution mapping, embryos from natural matings are preferable (right column). To define the interval containing the mutation, polymorphisms derived from the meiotic map ~3-10cM apart flanking the locus are [file NIHMS148281-supplement-04.tif]
