# Supplementary material for: Absence of heartbeat in the Xenopus tropicalis mutation muzak is caused by a nonsense mutation in cardiac myosin myh6
Source: Dev Biol. Author manuscript; Available in PMC 2010 Dec 1. (PMC2786259; doi:10.1016/j.ydbio.2009.09.019)
Supplement: 02 — Table S1. Gene models in the muz-containing genetic interval and their official full names as provided by the HUGO gene nomenclature committee. [file NIHMS148281-supplement-02.doc]

**Table S1.** Gene models in the *muz*-containing genetic interval and their official full names as provided by the HUGO gene nomenclature committee.

| **Symbol** | **Name** |
| --- | --- |
| myh6.2 | myosin heavy chain 6.2 |
| myh6 | myosin heavy chain 6 |
| efs | embryonal Fyn-associated substrate |
| pabp2 | poly (A)-binding protein 2 |
| bcl2l2 | BCL2-like 2 |
| ppr3d | phosphatase 1 regulatory subunit 3D |
| twsg2 | twisted gastrulation homolog 2 |
| homez | homeobox and leucine zipper encoding |
| slc7a8 | solute carrier family 7 (cationic amino acid transporter, y+ system), member 8 |
| cebpe | CCAAT/enhancer binding protein (C/EBP), epsilon |
| pck2 | phosphoenolpyruvate carboxykinase 2 (mitochondrial) |
| wdr23 | WD repeat domain 23 |
